# Experimental Insight into Thermally Driven Structural Evolution and Metastable Dynamics of Cholesteric Liquid Crystal Droplets

**DOI:** 10.3390/molecules31183357

**Published:** 2026-09-21

**Authors:** Shuting Xie, Haopeng Zhang, Lu Jiang, Hua Zhang, Peiwang Li, Aihua Zhang, Changzhu Li, Zhihong Xiao

**Affiliations:** 1 State Key Laboratory of Woody Oil Resources Utilization, Hunan Academy of Forestry, Changsha 410004, China; 2 Joint Laboratory of Optofluidic Technology and Systems, South China Academy of Advanced Optoelectronic, South China Normal University, Guangzhou 510006, China; 3 International Academy of Optoelectronics at Zhaoqing, South China Normal University, Zhaoqing 526238, China

**Keywords:** cholesteric liquid crystals, microdroplets, phase transition, thermally responsive, self-assembly

## Abstract

Understanding the thermo-responsive behavior of confined liquid crystals is essential for advancing their use in functional photonic devices. In this study, the temperature-driven phase transitions, molecular arrangements, and intermediate structural states of cholesteric liquid crystals (CLCs) confined within microdroplets are investigated. Monodispersed CLC droplets exhibiting a stable radial-spherical-structure (RSS) are fabricated via microfluidics to explore continuous transitions between highly ordered helical architectures and disordered isotropic phases. During heating, localized distortions appear within the concentric-ring pattern of the RSS texture, followed by ordered domains shrinking as the CLC becomes isotropic. This behavior could be mainly attributed to the fact that the system overcomes the free-energy barrier stabilizing the RSS configuration once the temperature is raised to 54 °C. Interestingly, the change process of CLC during cooling is not entirely the reverse process of the heating process. The “radical” multidomain and the blue phase-like texture are sequentially formed during the process from isotropic to cholesteric. Furthermore, faster cooling rates directly increase nucleation density and accelerate tactoid growth. In addition, the elimination of tactoid boundaries during nucleation and self-assembly enables the formation of large aggregates, which ultimately relax back to the stable RSS configuration driven by overall free-energy minimization. These experimental insights into the spatially confined self-assembly and thermo-responsive kinetics of CLCs provide a critical foundation for advancing their application in sensing, displays, and functional photonic devices.

## 1. Introduction

Cholesteric liquid crystals (CLCs) are widely recognized for their periodic rotational alignment of molecules along a helical axis, forming self-assembled structures with characteristic pitch lengths [1]. This unique property has drawn significant attention in fields such as optical modulation [2,3], soft matter topological design [4], and smart responsive materials [5,6]. In recent years, confining CLCs within microscale droplets has emerged as a prominent research direction [7,8]. As a typical finite boundary system, droplets not only introduce substantial surface anchoring effects but also exhibit rich topological structures and complex non-equilibrium dynamic behaviors [9,10,11]. The structure of CLC droplets could be continuously transformed from a twisted bipolar structure (TBS) to a radial spherical structure (RSS) as the chirality of LC confined in micron-sized droplets increases, which was investigated by a combination of theory and experiment [12,13]. This structural transition is attributed to the balance among liquid crystal elastic properties, chiral interactions and surface energy in confined space.

Temperature, as one of the most fundamental and effective external control parameters, plays a decisive role in the alignment of LC molecules within the droplet and serves as a crucial means to regulate its structure and function [4,14]. Due to the presence of boundary confinement effects, CLCs within droplets cannot form an ideal infinite-period spiral structure. Instead, they release elastic energy by introducing topological defects (such as boojums, hedgehogs, and torons) [15]. The spatial distribution and stability of these defects are highly dependent on temperature [16,17]. Periodic fingerprint texture or multi-ring structures of the CLC droplets are usually formed at low temperatures due to a short pitch and strong elastic anisotropy [14,18]. As the temperature increases, the original periodic stripes gradually break and evolve into localized defect structures, such as torons or single-point defect states because the system tends to minimize its torsional energy [9,19]. During this process, the generation, migration, and annihilation of defects are the dominant dynamic processes, reflecting the system’s transition behavior between different energy minima.

As the temperature further increases and approaches the clear point, CLC gradually undergoes a phase transition from an ordered cholesteric phase to a nematic phase, ultimately entering an isotropic liquid state [12,20]. During this process, the spiral structure is completely unrolled, and topological defects gradually disappear. The optical anisotropy inside the droplet is significantly reduced or even completely eliminated [21,22]. In addition, non-equilibrium dynamic processes are also an important frontier direction in current research [23]. Recent studies have no longer been limited to steady-state structural analysis but increasingly focus on the influence of temperature change rate on the pathways of structural evolution [24,25]. For example, the system tends to gradually approach an equilibrium state under slow thermal regulation [26,27]. However, the intermediate state structures and dynamic processes involved in the continuous transition from highly ordered helical structures to disordered isotropic phases are still unclear.

In this study, the temperature-driven phase transition process and molecular arrangement of CLCs in the confined space of microdroplets is investigated. The intermediate structural states and dynamic processes involved in the continuous transition from highly ordered helical structures to disordered isotropic phases or the opposite process are experimentally explored. Additionally, the detailed nucleation and growth kinetics of LC domains during the transformation from the isotropic to cholesteric phase in droplets is discussed. This temperature-driven phase transition process exhibits significant path dependence and hysteresis within droplet systems, where the structural evolution pathways during heating and cooling are not completely reversible, reflecting complex non-equilibrium thermodynamic characteristics. Understanding the regulation of temperature in the phase transition process and LC molecular arrangement of CLC within droplets will facilitate the advanced application of CLCs in functional devices.

## 2. Results and Discussions

### 2.1. The Temperature Controllable Stage and CLC Droplets

The cholesteric liquid crystal (CLC) used in the experiment is obtained by adding 1.0 wt% chiral agent molecule S1011 to E7, a nematic liquid crystal (LC). The chemical structures of every component in the microfluidic generation of CLC droplets are shown in Figure S2. A 3.0 wt% PVA aqueous solution is selected as the external phase and CLC is the internal phase. They are, respectively, injected into the corresponding microchannels in the hydrophilically modified PDMS chip, and then droplets with uniform size and controllable size are obtained under the joint action of the shear force and viscosity of the fluid (Figure S2d). When the volume flow rates of the two phases are, respectively, 8.0 μL/min and 0.2 μL/min, the diameter of the obtained droplet is ~65 μm.

**Figure 1:**
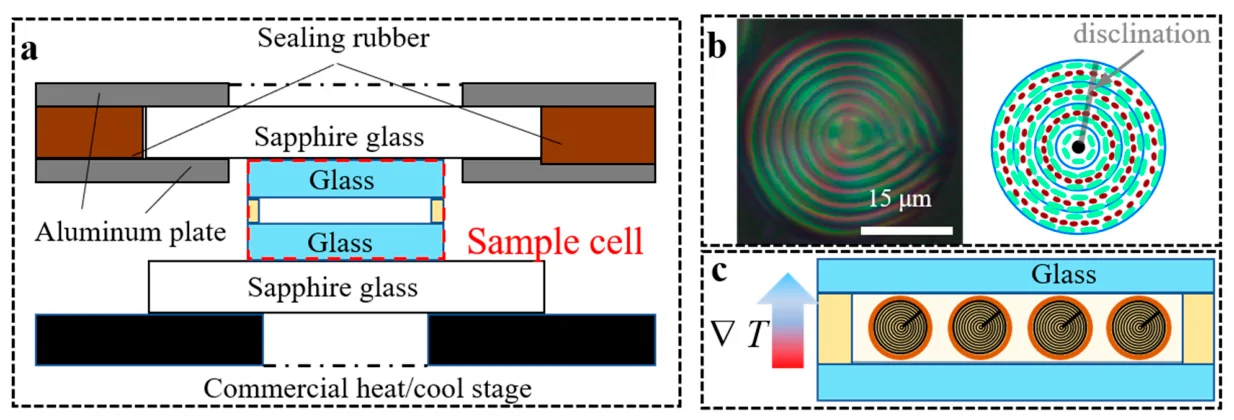
(**a**) Schematic illustration of temperature controlling plate of Linkam Heating Table in experiments. (**b**) POM image and schematic drawing of a CLC droplet with RSS structure. (**c**) Temperature gradient of a homemade device with LC droplets within it.

To observe the structural evolution of the CLC droplets under the regulation of temperature, the collected droplets are transferred to a custom-built observation device and then positioned on a Peltier-based temperature-controlled stage, which was supported by a commercial Linkam Heating Table, as shown by the schematic in Figure 1a. It enabled both heating and cooling of the CLC droplets to a controlled desired temperature during the optical measurements. A highly ordered concentric and spiral-like pattern with periodic rings, namely, a radial spherical structure (RSS), is observed under polarized mode (the left image of Figure 1b). The formation of the RSS structure is attributed to the combined effects of PVA-induced interfacial anchoring and three-dimensional confinement [28]. On the basis of the infinitely strong tangential anchoring of PVA, spherical packing of CLC layers is confined in a concentric manner with the normal m to a radical point-defect hedgehog with the core in the droplet center. These point defects simultaneously form radial defects—disclinations—in the local director field n under the effect of spherical confinement [6,28]. The presence of well-defined circular fringes indicates a three-dimensional periodic structure with submicron lattice spacing comparable to visible wavelengths, leading to strong Bragg reflection [29,30]. The spacing between adjacent texture bands reflects the cholesteric pitch, which is measured to be 2.3 μm, in good agreement with the theoretical value of 2.5 μm [12]. As shown in Figure 1c, the temperature of the observation device would decrease along the direction from the bottom substrate to the top substrate during the heating process.

### 2.2. Effect of Heating on the Molecular Arrangement of LCs

**Figure 2:**
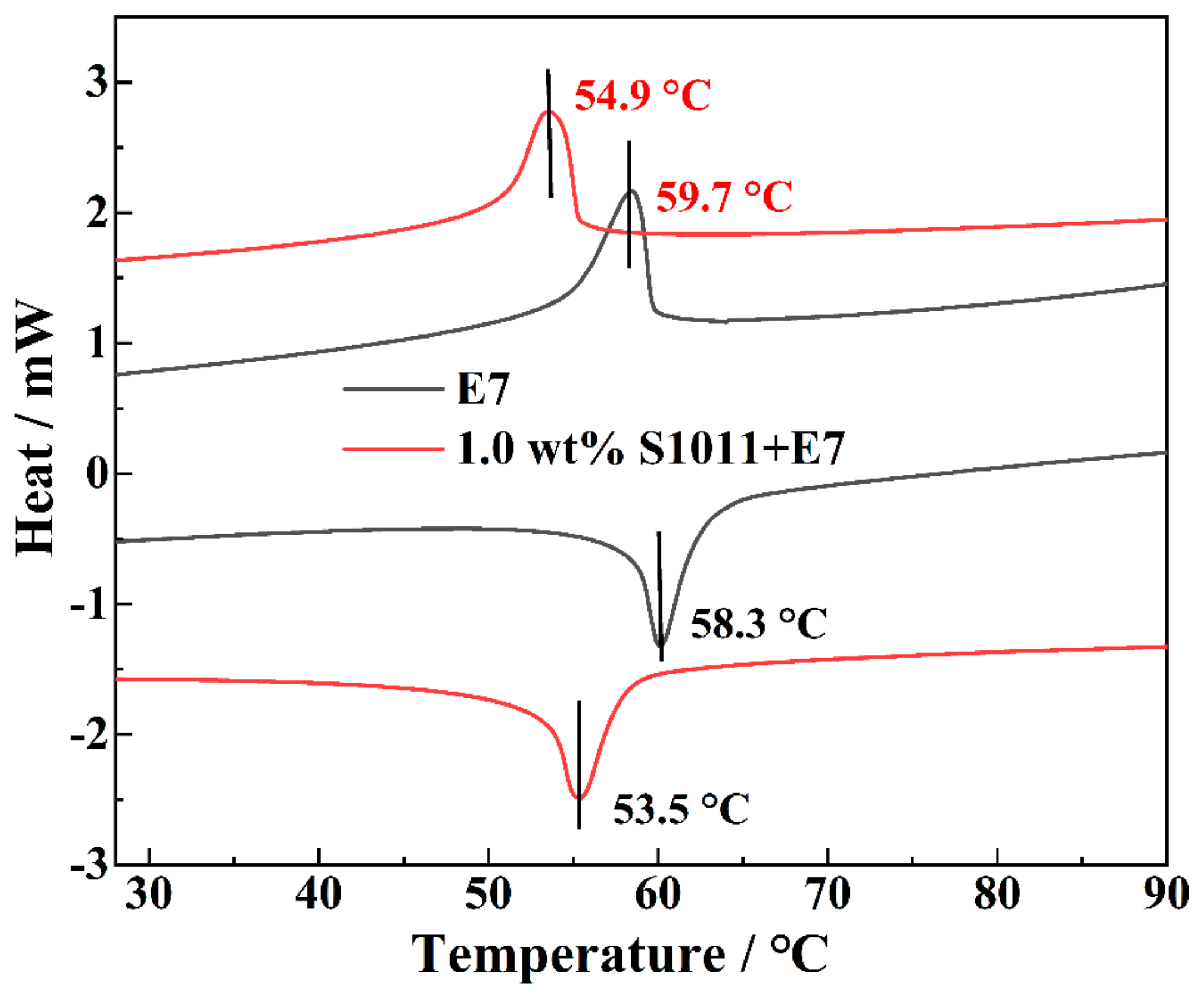
DSC curves of different liquid crystals.

Thermotropic LCs undergo temperature-driven phase transitions, typically evolving from an anisotropic liquid-crystalline state to an isotropic liquid upon heating. Because these transitions are accompanied by pronounced changes in long-range molecular order, the phase-transition temperature is a key parameter for characterizing thermotropic LCs. Accordingly, the transition temperatures of the CLC used in this study were determined by differential scanning calorimetry (DSC), as shown in Figure 2. Upon heating, pure E7 and CLC (1.0% S1011 + E7) exhibit endothermic peaks at 59.7 °C and 58.3 °C, respectively, corresponding to the LC-to-isotropic transition temperature (T_NI,heating_). Upon cooling, the reverse isotropic-to-liquid-crystalline transition occurs at 54.9 °C for E7 and 53.5 °C for CLC, which is the value of T_NI,cooling_. T_NI,cooling_ is approximately 5 °C lower than T_NI,heating_, indicating that introducing a chiral dopant not only alters the phase behavior of the LC but also lowers its phase-transition temperature.

**Figure 3:**
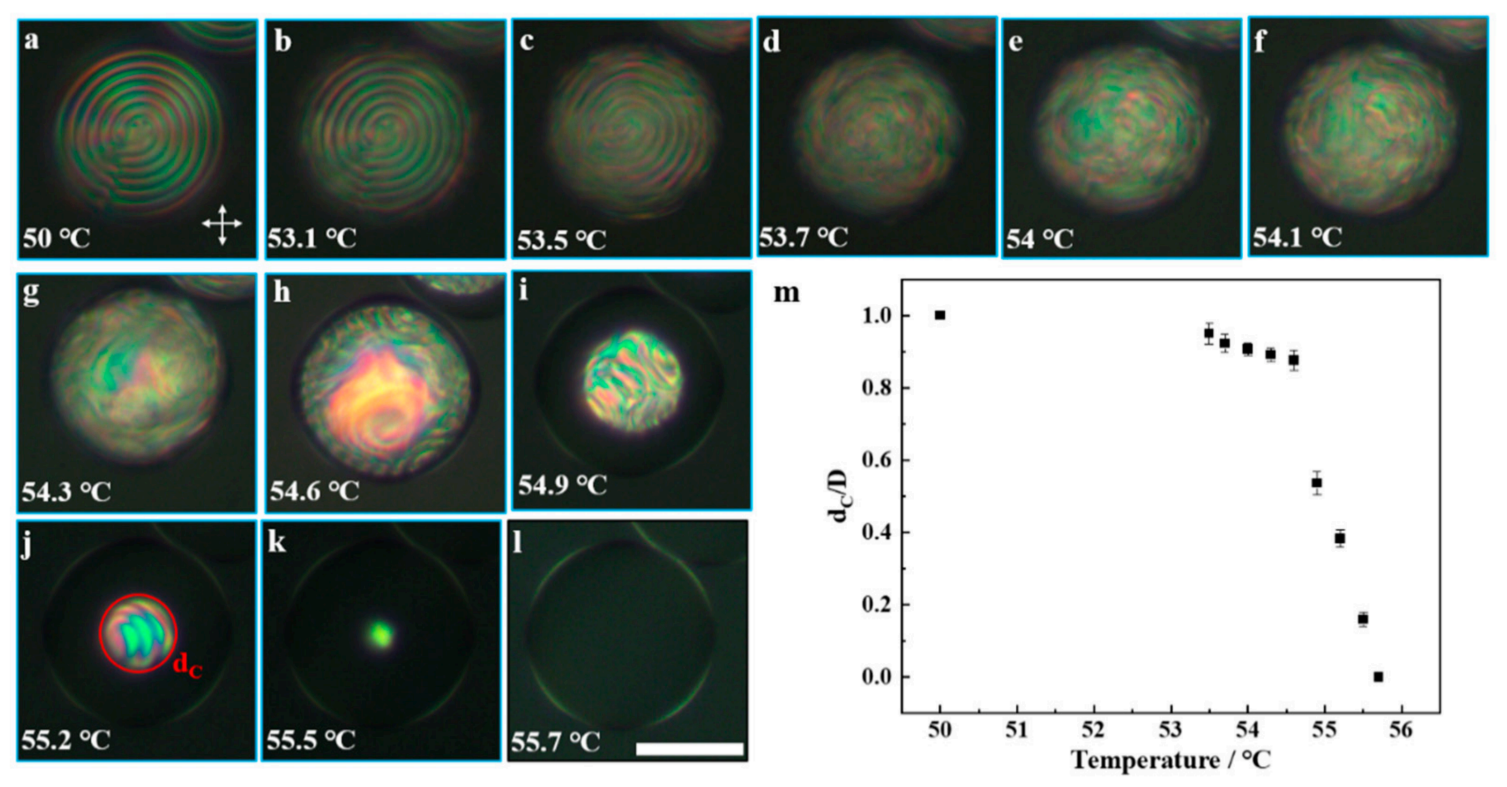
Effect of heating on the molecular arrangement of CLC. (**a**–**l**) POM images of a CLC droplet at a series of temperatures during the heating process. POM images are obtained under orthogonal polarization condition which is exhibited by the white cross in (a) The scale bar in the pictures represents 15 μm. (**a**) 50 °C, (**b**) 53.1 °C, (**c**) 53.5 °C, (**d**) 53.7 °C, (**e**) 54 °C, (**f**) 54.1 °C, (**g**) 54.3 °C, (**h**) 54.6 °C, (**i**) 54.9 °C, (**j**) 55.2 °C, (**k**) 55.5 °C, and (**l**) 55.7 °C. (**m**) A plot of d_c_/D versus temperature, showing the cholesteric-to-isotropic phase transition. d_c_/D is analyzed from Supplementary Movie S1 showing heating on the structural evolution of 6 independent CLC droplets with comparable diameters.

To probe the thermal evolution of molecular alignment within the droplets, CLC droplets initially equilibrated at 25.0 °C are heated to 60.0 °C at a rate of 5 °C min^−1^. It should be noted that pressure may also influence the phase transition behavior of CLCs by modifying molecular packing and helical ordering [9]. In this work, all measurements were conducted under atmospheric pressure, and therefore the observed temperature-dependent nucleation and structural evolution mainly reflect thermal effects under constant pressure conditions. During heating, the organization of internal LC molecules progresses through three steps: an essentially unchanged RSS texture, progressive disordering of the molecular arrangement, and the cholesteric-to-isotropic phase transition (Figure 3, Supplementary Movie S1). When the temperature is in the range of 25.0–56.0 °C, no discernible change in the POM texture is observed, indicating that the droplets retained the RSS configuration (Figure 3a). At 53.5 °C, however, localized distortions appear within the concentric-ring pattern of the RSS texture (Figure 3c), indicating the onset of realignment. Consistent with this observation, the POM images in Figure 3b–f show a continuous evolution of the molecular arrangement between 53.1 and 54.1 °C. This behavior can be mainly attributed to the fact that the system overcomes the free-energy barrier stabilizing the RSS configuration once the temperature is raised to 53 °C [31]. The enhanced Brownian motion of the LC molecules then promotes their rearrangements, leading to pronounced changes in RSS structure [31]. At 54.3 °C (Figure 3g), small dark domains first appear near the LC–water interface, suggesting the onset of an isotropic state in the near-surface region. With further heating (Figure 3i–k), these dark domains expand and propagate inward from the droplet periphery toward the core. At 55.7 °C (Figure 3l), the droplet becomes uniformly dark under crossed polarizers, confirming that the LC has fully transitioned to the isotropic state. It should be noted that the POM-observed transition temperature shift relative to the DSC value is attributed to the different principles and sensitivities of these techniques. Overall, these observations indicate that increasing temperature enhances the molecular mobility (thermal fluctuations) of the LC molecules. Figure 3m is a plot of dc/D versus temperature, showing the cholesteric-to-isotropic phase transition. Once the temperature exceeds ~53 °C, dc/D is smaller than 1.0, indicating that RSS becomes progressively destabilized and gradually disrupted. When the temperature reaches T_NI,heating_ (54.9 °C, Figure 2), the LC undergoes a complete transition to the isotropic phase, where long-range orientational order is finally lost and the molecular arrangement becomes disordered.

### 2.3. Effect of Cooling on Molecular Alignment of CLCs

**Figure 4:**
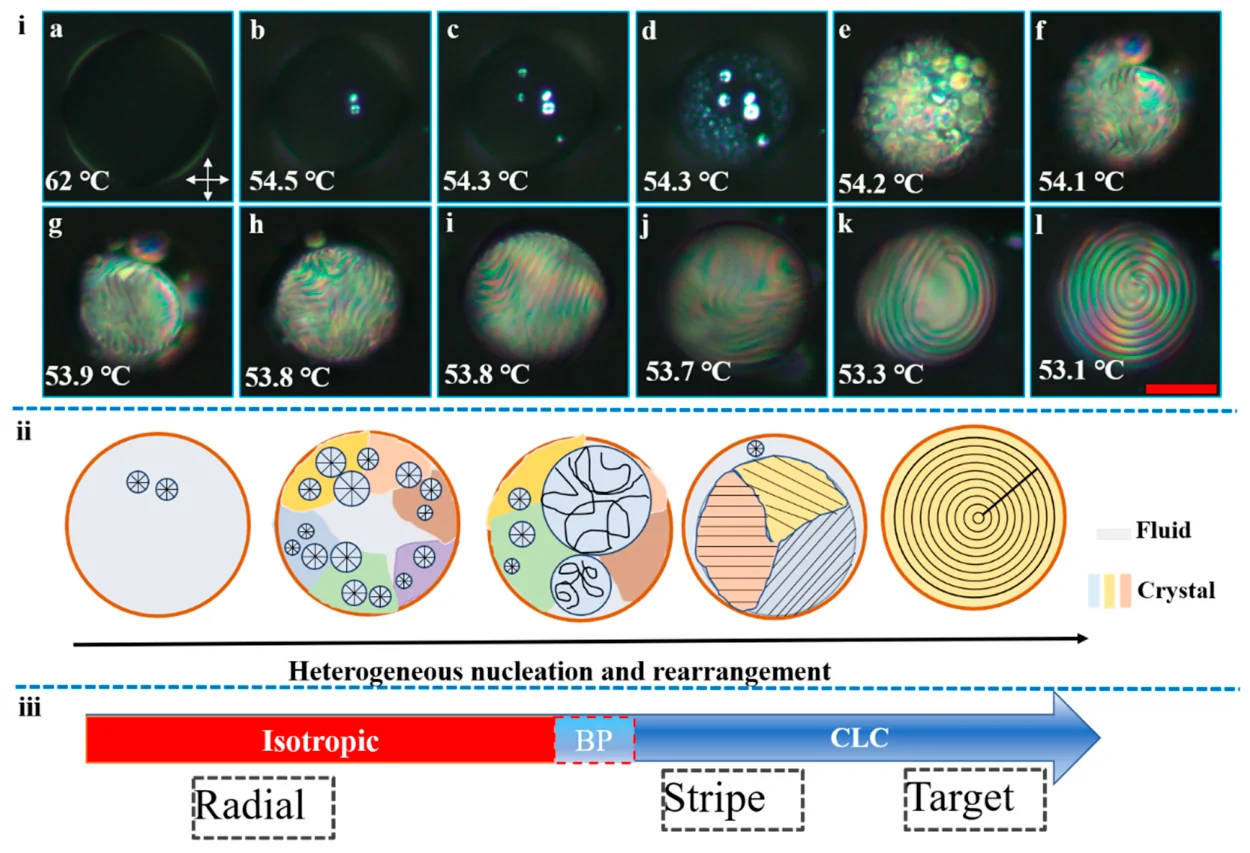
Molecular alignment of CLC droplet during the cooling process. (**i**) POM images of CLC droplet at a series of temperatures during cooling with a cooling rate of 0.8 °C/min. (**a**) 62 °C, (**b**) 54.5 °C, (**c**) 54.3 °C, (**d**) 54.3 °C, (**e**) 54.2 °C, (**f**) 54.1 °C, (**g**) 53.9 °C, (**h**) 53.8 °C, (**i**) 53.8 °C, (**j**) 53.7 °C, (**k**) 53.3 °C, and (**l**) 53.1 °C. The scale bar in the pictures represents 15 μm. The white cross in (**a**) represents the orthogonal polarization condition. (**i**,**ii**) Schematic illustration of (**ii**) the heterogeneous nucleation and rearrangement of LC molecules and (**iii**) the phase change under the cooling process. The different colors in the CLC sphere shown in (**i**) represents different domains.

After the CLC had transitioned to the isotropic state, the sample is cooled from 62.0 °C to 50.0 °C at 0.8 °C min^−1^, while the phase state and structural evolution are also monitored in real time by POM. During cooling, the system gradually evolves from a disordered isotropic phase to an ordered liquid-crystalline phase. Figure 4 and Supplementary Movie S1 shows several interesting features observed during this self-assembly process. As the temperature decreases from 62.0 °C to 54.5 °C (Figure 4a), one or two small blue domains emerge inside the droplet. These domains exhibit a well-defined dark Maltese cross pattern, which is characteristic of a radial configuration. By comparison with prior reports [32], these radially textured blue domains are assigned to the blue phase (BP)-like state based on the characteristic optical textures and phase behavior. Due to the small droplet size and extremely narrow temperature range, current optical evidence cannot completely rule out other complex anomalies, and further confirmation is needed through laser confocal or reflection spectra. Upon further cooling (Figure 4b–d), the BP domains grow and progressively coalesce. At 54.2 °C (Figure 4e), the droplet contained numerous domains spanning a broad size distribution, most of which appears yellow. When the temperature is reduced slightly to 54.1 °C (Figure 4f), these domains merge into larger hybrid regions, within which stripe-like textures begin to emerge locally. Upon further cooling to 53.9 °C (Figure 4g–i), the domains formed at earlier stages had largely coalesced. This coalescence is accompanied by pronounced molecular reorganization within the LC, reflecting a continuous relaxation toward a lower-energy configuration. When the temperature reaches 53.1 °C (Figure 4l), the texture evolves into a well-defined RSS, in full agreement with the RSS configuration shown in Figure 1b.

Throughout the cooling process, the assembly of LC molecules within the microdroplets during the isotropic-to-liquid-crystalline transition is not entirely the reverse process of the heating process, which could be rationalized as follows. As the temperature approaches T_NI,cooling_, the isotropic fluid begins to nucleate an ordered phase, giving rise to tactoids. These tactoids are analogous to nuclei formed during conventional crystallization, which are commonly regarded as “LC nuclei”. Accordingly, the small radially textured domains observed in Figure 4b are assigned to nuclei generated during the isotropic-to-liquid-crystalline phase transition [24]. With further cooling, these nuclei grow and progressively coalesce. Because the temperature within a droplet is not perfectly uniform, local nucleation and growth rates can vary spatially, leading to domains with different characteristic sizes and optical colors. As growth proceeds, coalescence among neighboring domains produces hybrid domains separated by transient interfaces. The associated interfacial energy can strongly influence subsequent growth and coarsening. Driven by overall free-energy minimization [29], the LC molecules undergo concomitant self-reorganization during domain fusion, which promotes the emergence of increasingly ordered, stripe-like textures. Ultimately, these stripe patterns evolve into an RSS structure under the combined influence of energetic relaxation and the interfacial constraints imposed by PVA [26,33]. Figure 4ii provides a corresponding schematic overview of the nucleation-and-growth process and the corresponding molecular self-assembly behavior of CLC throughout cooling. The phase sequence observed upon cooling is summarized in Figure 4iii, which shows a stepwise transition from the isotropic phase to the blue phase-like state and subsequently, to the cholesteric phase. Notably, the blue phase-like state is stable over a narrow temperature window of 54.5–54.3 °C.

To further elucidate the influence of confinement geometry on nucleus growth and molecular alignment, the LC is also examined under quasi-two-dimensional confinement in a planar LC cell. The sample is cooled from 62.0 °C to 50.0 °C at the same rate, while the phase state and assembly dynamics are monitored in real time by POM. As shown in Figure S3, the overall phase evolution and alignment behavior in the planar cell are broadly consistent with those observed in spherical droplets. However, there are two key differences. Firstly, the characteristic temperatures associated with each transition stage are slightly shifted. Secondly, after small domains merge, the phase boundaries in the planar cell remain more sharply defined than those in droplets, suggesting differences in interfacial energetics and/or relaxation pathways under planar confinement. This observation is consistent with the phase sequence inferred from the droplet experiments.

During the cooling process, the molecular alignment of the LC—as well as the nucleation, emergence, and subsequent growth of mesoscopic domains—is primarily regulated by the anisotropic nematic (N)–isotropic (I) interface [34,35]. The interfacial energy mainly consists of two contributions: the surface energy (*F*_s_) and the bulk free-energy difference between the isotropic and nematic phases (*f*_I_ − *f*_N_). The surface energy *F*_s_ is defined as follows:

(1) $$F_{s}\;=\;2\pi \sigma_{0}r_{1/2}$$

Here, *σ*_0_ denotes the surface tension of LC, and *r*_1/2_ represents the critical nucleus size. The aggregation energy (*f*_I_ − *f*_N_) is proportional to the entropy of the system and its temperature variation [36]:

(2) $$\left(f_{I}\;-\;f_{N}\right)\pi r_{1/2}^{2}\propto \left(S_{I}-S_{N}\right)\left(T_{\mathrm{NI}}-T\right)r_{1/2}^{2}$$

Here, *S*_I_ and *S*_N_, respectively, denote the entropies of the liquid crystal in the isotropic and nematic states.

When the isotropic liquid coexists with the liquid crystalline phase, the critical nucleus size *r*_1/2_ is [24]

(3) $$r_{1/2}=\frac{1}{f_{I}-f_{N}}\left[\sqrt{\sigma_{1/2}^{2}+\frac{k}{2}\cdot (f_{I}-f_{N})}-\sigma_{1/2}\right]$$

Here, *k* is a constant, and *σ*_1/2_ denotes the anisotropic interfacial energy.

According to the above equations, the characteristic nucleus size varies systematically with temperature and the nucleation rate is governed by the degree of undercooling, i.e., the temperature difference between the transition temperature and the system temperature (*T*_NI_ − *T*). Guided by this theoretical framework, it is necessary to investigate how the cooling rate influences molecular ordering and the phase-transition crystallization of LC confined within droplets.

### 2.4. Effect of Cooling Rate on the Crystallization and Molecular Alignment of LC

**Figure 5:**
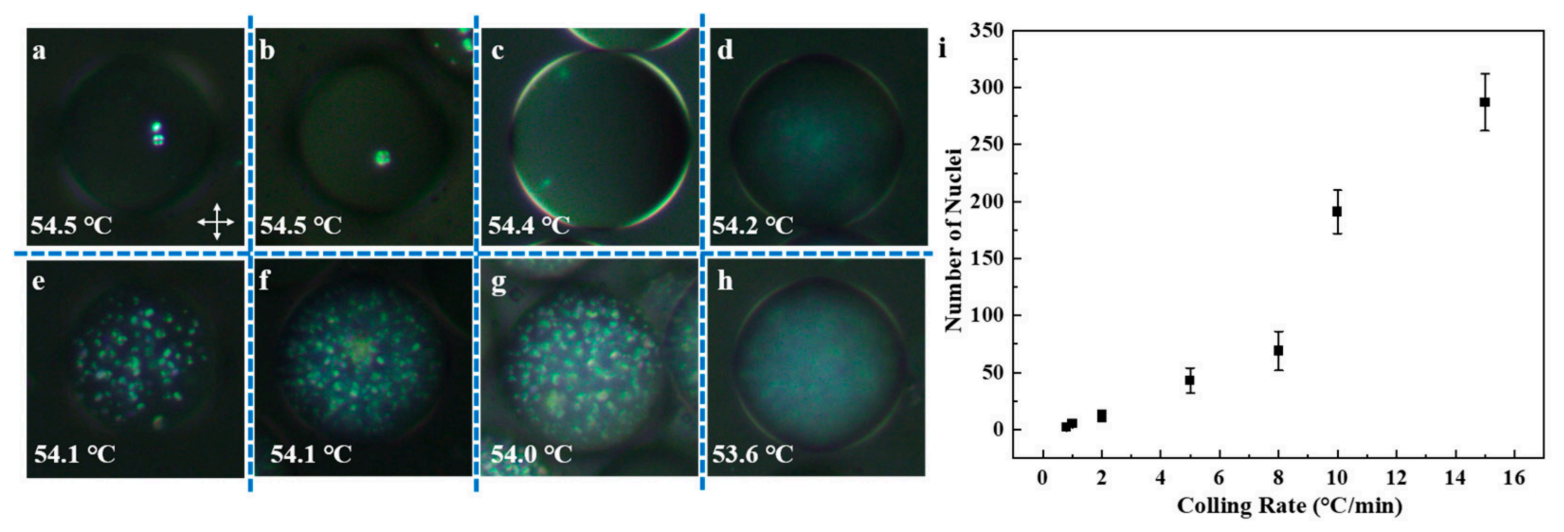
The POM images of an LC droplet when it was cooled from 55 °C to ~53 °C at different cooling rates: (**a**) 0.8 °C/min, (**b**) 1 °C/min, (**c**) 2 °C/min, (**d**) 5 °C/min, (**e**) 8 °C/min, (**f**) 10 °C/min, (**g**) 15 °C/min, and (**h**) 20 °C/min. The scale bar in the pictures represents 15 μm. The white cross in (**a**) represents the orthogonal polarization condition. (**i**) The number of nuclei plotted as a function of cooling rate. The numbers of nuclei were extracted from the movies of CLC droplets cooled from 55 °C to ~53 °C at different cooling rates when temperature = 54.1 °C.

After the LC transitioned to the isotropic state, the sample is cooled from 56.0 °C to 54.0 °C at different cooling rates (Figure 5). As seen in Figure 5a, two bright spots assigned to nuclei formed inside the droplet during the isotropic-to-liquid-crystalline transition when cooling rate = 0.8 °C min^−1^. Under POM, these nuclei exhibit a radial texture, identical to the optical texture observed for nematic droplets under homeotropic anchoring. When the cooling rate = 1 °C min^−1^ (Figure 5b), a single radial nucleus is also observed, while its size is noticeably larger than either of the two nuclei in Figure 5a. At higher cooling rates of 2 °C min^−1^ (Figure 5c) and 5 °C min^−1^ (Figure 5d), the number of nuclei increases markedly. With further increases in the cooling rate (Figure 5e–h), the number of nuclei formed per unit time rises significantly. However, the microscopic textures of these small nuclei become indistinct, and they are mainly observed as blue features. These results indicate that the number of crystallization sites is strongly dependent on the cooling rate. The number of nuclei were extracted from the movies of LC droplets cooled from 55 °C to ~53 °C at different cooling rates when temperature = 54.1 °C and plotted as a function of cooling rate (Figure 5i). The analysis reveals that number of nuclei exhibits a clear dependence on the cooling rate. The number of nuclei shows a significant increase with the increase in cooling rate, indicating that an increase in the cooling rate could significantly promote the nucleation process. According to Equation 2, classical homogeneous nucleation theory [24], the degree of supercooling (*T*_NI_ − *T*) plays a key role in the nuclei process. When rapidly cooling down, the system does not have time to undergo significant crystallization at higher temperatures and will enter a deeper supercooled state. So the instantaneous nucleation rate may increase at a higher cooling rate, producing more nuclei within a given time window. Moreover, the aggregation energy (*f*_I_ − *f*_N_) is also proportional to the degree of supercooling (*T*_NI_ − *T*). Thus, rapid cooling also leads to smaller nucleus sizes, which is evidenced by an indistinguishable nucleus.

### 2.5. Growth of LC Crystals and Their Molecular Alignment

**Figure 6:**
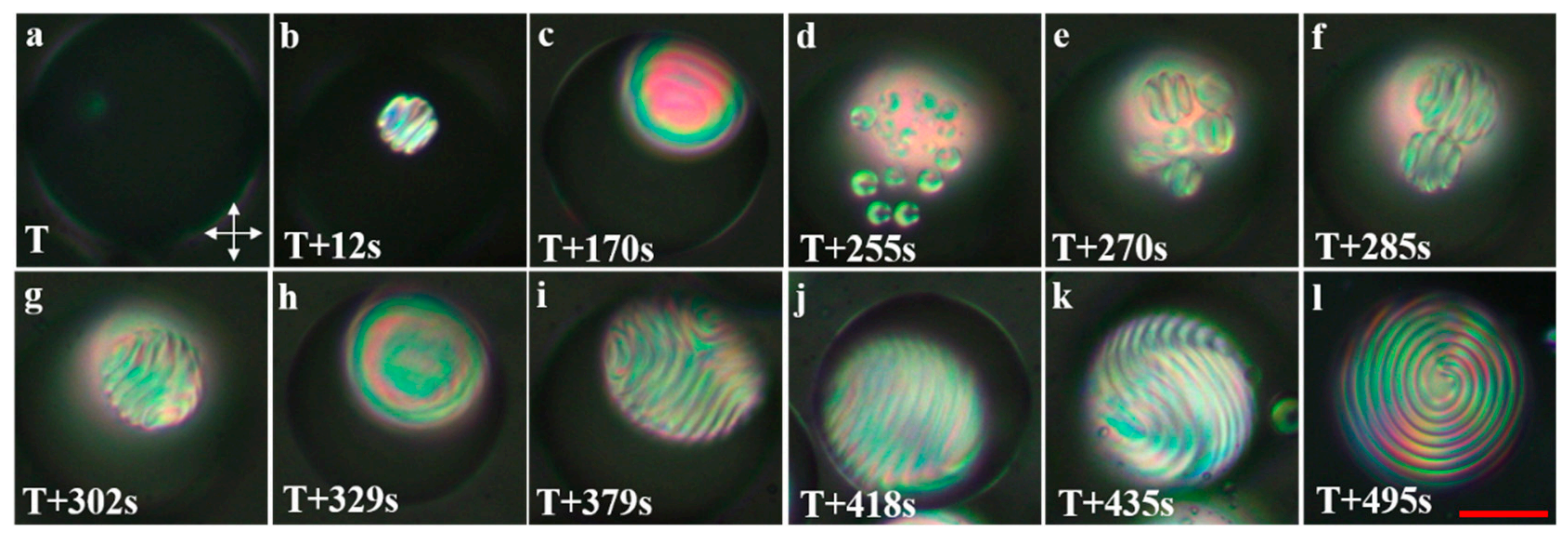
The POM images of a CLC droplet when it was kept at 53 °C at different moments. (**a**) T, (**b**) T+12 s, (**c**) T+170 s, (**d**) T+255 s, (**e**) T+270 s, (**f**) T+285 s, (**g**) T+302 s, (**h**) T+329 s, (**i**) T+379 s, (**j**) T+418 s, (**k**) T+435 s, (**l**) T+495 s.T was defined as the moment when the crystal nucleus just appeared. The white cross in (**a**) represents the orthogonal polarization condition.The isotropic CLC droplets were firstly obtained by heating to 55 °C at 0.8 °C/min and then cooling to 53 °C at 0.8 °C/min. The scale bars in the pictures represent 15 μm.

After nucleation, the question arises as to how the LC molecules inside the droplet recover the RSS configuration. To elucidate the intermediate pathway, droplets that are initially in the isotropic state are held at 53.0 °C on a temperature-controlled stage for 10 min. Figure 6 and Supplementary Movie S2 show representative POM micrographs recorded during the isothermal process. The moment at which a nucleus first appeared is defined as time T. At T (Figure 6a), a faint bright spot corresponding to the nucleus is visible within the otherwise dark droplet. It represents the crossing of the thermodynamic energy barrier in the crystallization process, leading to primary nucleation. The nuclei begin to spontaneously grow outward as time goes by. After 12 s (Figure 6b), the nucleus gradually grows into a tactoid aggregate and exhibits a helical texture. After 170 s (Figure 6c), the nucleus continues to grow and appears red. After 255 s (Figure 6d), numerous small nuclei exhibiting a four-lobed (clover-like) texture reappear inside the droplet. With further time, nuclei with a radial configuration merge to form helical tactoid-like aggregates. These tactoid aggregates continue to grow and coalesce over time, ultimately producing larger tactoid domains (Figure 6e–h). It can be seen that there is a clear phase interface between the isotropic phase and LC phase/tactoid. When the elapsed time is sufficiently long (Figure 6i), LC molecules within the large aggregates reorganize toward a lower free-energy state, giving rise to a fingerprint-like texture with quasi-concentric rings. As shown in Figure 6k, as long as the system has not fully transformed into the liquid-crystalline phase, nucleation continues to occur. The above sequence then repeats until all molecules in the droplet convert from the isotropic state to the liquid-crystalline state and reassemble into the same RSS configuration, as illustrated in Figure 6l. The crystallization at 53 °C indicates that the temperature is below T_NI_ of the CLC; thus, a certain degree of undercooling exists in the system. The undercooling provides the thermodynamic driving force required for the phase transition. The sudden appearance of a crystal nucleus at T proves that the fluctuations in the thermal motion of LC molecules are sufficient to overcome the nucleation barrier and form stable crystal nuclei that reach the critical size at 53 °C.

## 3. Materials and Methods

### 3.1. Materials

Nematic liquid crystal E7 (99%) and chiral agent S1011 (≥95%) were purchased from Jiangsu Hecheng Display Technology Co., Ltd. (Suzhou, Jiangsu, China). Polyvinyl alcohol (PVA, Mn = 13,000 g/mol) was obtained from Sigma Aldrich Reagent Co., Ltd. (Shanghai, China). Anhydrous ethanol (EtOH, ~99.5%) was from Guangzhou Reagent Co., Ltd. (Guangzhou, Guangdong, China). The deionized water (18.25 mΩ·cm, 25 °C) used in the experiment was prepared by the laboratory pure water manufacturing system (wp-up-uv-20, Sichuan wattle water treatment equipment Co., Ltd., Chengdu, China).

### 3.2. Preparation of CLC Droplets

The CLC droplets were formed using the method previously reported [6,37]. The aqueous phase used in the experiment is 3.0 wt% PVA aqueous solution, and the oil phase is CLC prepared by E7 doped with 1.0 wt% S1011. The phase transition temperature of E7 and CLC is characterized through a differential scanning calorimeter (DSC1, Mettler Toledo, Greifensee, Switzerland) to measure the endothermic and exothermic curves in the temperature scale of −20–120 °C at a temperature rise and fall rate of 2 °C/min. The flow-focusing polydimethylsiloxane (PDMS) microfluidic chip used was self-obtained by soft-lithography and partial channels are hydrophilically modified by N-isopropylacrylamide (NIPPAM) (details are provided in Supplementary Information). The two phases are, respectively, injected into the corresponding channel in the microfluidic chip by a pressure injection pump (TYD01-01, Ralph fluid equipment Co., Ltd., Baoding, Hebei, China). To obtain CLC droplets with tunable size, the flow rates of the fluids are controlled, respectively, in the range of 0.2–10 μL/min. The prepared emulsion droplets were collected in a 5 mL gray vial for observing the size and texture of the droplets.

### 3.3. Texture of CLC Droplet During the Temperature Control of Emulsion

The collected CLC droplets within a self-made 3 cm × 3 cm × 100 μm observation device were placed on a Linkam Heating Table (LTSE420, Linkam Cooling and Heating Table Co., Ltd., Salfords, UK) to control the temperature (Figure S1). This is a high-precision temperature control system equipped with a temperature sensor and feedback regulation module. The heating/cooling process was regulated through a feedback loop to maintain a stable temperature environment. The temperature controller provides a resolution of 0.01 °C, allowing precise adjustment and stabilization of the sample temperature. To observe the arrangement of LC molecules within the droplets during the heating or cooling process, the rate of temperature change was controlled. Simultaneously, CLC droplets were characterized by a polarizing microscope (Leica, DM2700p, Leica, Wetzlar, Germany) in transmission mode and recorded by a color CCD (Lumenera infinity 3-1, Lumenera, Ottawa, Canada) for their texture.

### 3.4. Characterization of Emulsion System Performance

The phase transition temperatures of E7 and CLC were measured by differential scanning calorimetry (DSC, Mettler-Toledo, Greifensee, Switzerland). Samples were subjected to heating and cooling runs at 2 °C min^−1^ over the range of −20 to 120 °C. The phase transition temperatures were determined from the recorded endothermic and exothermic thermograms.

The optical configurations of CLC droplets were examined using a polarized optical microscope (POM, Leica DM2700P, Leica, Wetzlar, Germany). Droplets were placed on the microscope stage and imaged under crossed polarizers using objectives of appropriate magnification. A color CCD camera (Lumenera Infinity 3-1, Lumenera, Ottawa, Canada) was used to capture and record the droplet textures and their temporal evolution.

## 4. Conclusions

In this work, the structural evolution and kinetic pathways underlying phase transitions of confined CLC droplets are elucidated, revealing the interplay between molecular ordering, thermal history, and self-assembly behavior. Monodisperse CLC droplets with RSS configurations are generated through droplet microfluidics, providing a well-defined platform for tracking temperature-dependent structural transformations. The confined cholesteric phase exhibits distinct thermal responses, where molecular organization and phase stability are strongly regulated by the competition between ordering tendency and thermal fluctuations. Systematic investigations further demonstrate that cooling conditions play a critical role in determining the nucleation behavior and subsequent hierarchical organization of cholesteric domains. The “radical” multidomain and the blue phase-like texture are sequentially formed during this process. A blue phase-like state is identified, while the final multidomain structures are governed by the balance between kinetic trapping and thermodynamic relaxation. During isothermal recovery, spontaneous nucleation and domain rearrangement drive the progressive reconstruction of helical architectures, leading to the restoration of stable RSS configurations through minimization of the system free energy. These findings provide fundamental insights into the self-assembly of LC molecules in spherical droplets and their thermo-responsive behavior, which may guide the development of LC-based applications in sensing, display, and related fields.

## Data Availability

The original contributions presented in this study are included in the article/Supplementary Material. Further inquiries can be directed to the corresponding author(s).

## Supplementary Materials

The following supporting information can be downloaded at https://www.mdpi.com/article/10.3390/molecules31183357/s1: Figure S1: A photo of Linkam Heating Table; Figure S2: The component of CLC and microfluidic formation of CLC droplets; Figure S3: A three-dimensional heat transfer model of a heating stage-homemade glass device. Figure S4: POM images of CLC film at a series of temperatures under the cooling process. Movie S1: CLC droplets under a cycle of heating and cooling. Movie S2: CLC droplets are kept at 53 °C.
