# Supplementary material for: Experimental Insight into Thermally Driven Structural Evolution and Metastable Dynamics of Cholesteric Liquid Crystal Droplets
Source: Molecules. 2026 Sep 21;31(18):3357. doi: 10.3390/molecules31183357 (PMC13610352; doi:10.3390/molecules31183357)
Supplement: Supplementary file 1 [file molecules-31-03357-s001.zip › supporting information.pdf]

Supporting Information for

Experimental Insight into Thermally Driven Structural Evolution and Metastable Dynamics of Cholesteric Liquid Crystal Droplets

Shuting Xie[1], Haopeng Zhang[2], Lu Jiang[1], Hua Zhang[3], Peiwang Li[1], Aihua Zhang[1], Changzhu Li[1*], Zhihong Xiao[1]*

[1] State Key Laboratory of Woody Oil Resources Utilization, Hunan Academy of Forestry, Changsha 410000, China

[2] Joint Laboratory of Optofluidic Technology and Systems, South China Academy of Advanced Optoelectronic, South China Normal University, Guangzhou 510006, China

[3] International Academy of Optoelectronics at Zhaoqing, South China Normal University, Zhaoqing 526238, China

† Authors to whom correspondence should be addressed: lichangzhu2013@aliyun.com ; xzhh1015@163.com.

Preparation of the droplet microfluidic chip by soft-lithography

The flow-focusing droplet-generation microfluidic chip was fabricated in polydimethylsiloxane (PDMS) using standard soft-lithography procedures. Briefly, the microchannel layout was designed to achieve the targeted droplet size, and a chrome photomask (0.5 μm resolution; Shenzhen Nanomei Chip Technology Co., Ltd., China) was prepared. A positive master mold was then produced on a silicon wafer by photolithography using SU-8 photoresist. After vapor-phase silanization of the patterned master with FDTS to render the surface hydrophobic, a PDMS mixture (prepolymer: curing agent = 10:1, w/w) was poured onto the master and thermally cured on a hot plate (EH20B, Beijing LabTech Co., China) at 90 °C for 30 min. The cured PDMS replica was peeled from the master, and inlet/outlet ports were created by punching. To assemble the device, the PDMS slab and a cleaned glass slide were treated with oxygen plasma (PDC-002, Harrick, USA) to generate surface hydroxyl groups and subsequently brought into conformal contact to achieve irreversible bonding, yielding a sealed chip capable of withstanding elevated operating pressures. For the generation of water-in–liquid-crystal droplets, the channel surfaces were further converted to hydrophilic. This hydrophilic modification was carried out by UV-induced graft polymerization of N-isopropylacrylamide (NIPAAm) on the PDMS surface.

Simulation of the temperature gradients across the observation device by COMSOL

A three-dimensional heat transfer model of a heating stage-homemade glass device is constructed firstly. The contact area between the hot stage and the bottom glass is set as a constant temperature heating boundary, while the central circular hole area does not directly contact the hot stage. Therefore, heat is mainly transmitted to the bottom glass from the contact area around the circular hole, and gradually transferred upwards through the heat conduction between the glass and the water layer. The metal glass interface uses thermal contact conditions to describe the interface temperature drop caused by actual contact. The outer surface of the device exposed to ambient air adopts

a natural convection boundary, and the ambient temperature is set to room temperature. Due to the small thickness of the water layer, the natural convection driven by buoyancy inside is significantly suppressed. Therefore, the model ignores fluid flow and considers the aqueous medium as a stationary fluid, with heat conduction as the main heat transfer mechanism. In steady state, the temperature field inside the device is determined by the thermal conductivity of each layer of material, the thermal resistance of the metal glass interface, and the heat dissipation from the surface to the air; Due to the lack of direct heating, the central pore area forms a local low-temperature zone, and the temperature diffuses from the surrounding hotter areas to the center, ultimately forming a stable three-dimensional temperature distribution with radial gradients in the water layer.

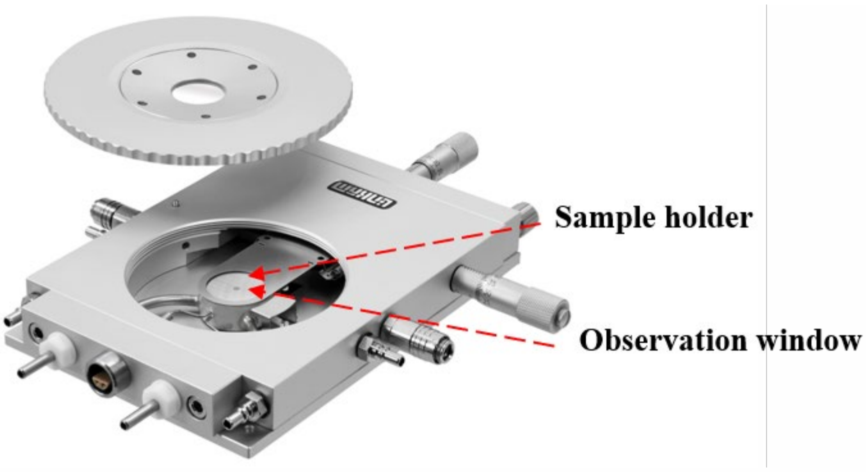

Fig. S1 A photo of Linkam Heating Table.

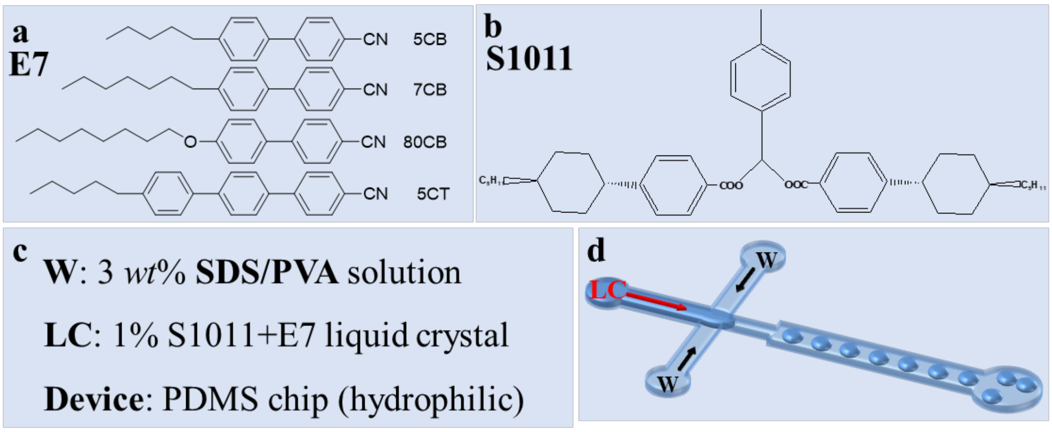

Figure S2 (a) The component of E7 and their molecular structures. (b) Molecular structure of chiral dopant S1011. (c) Fluids and device for droplet microfluidic systems. (d) Schematical illustration of droplet formation.

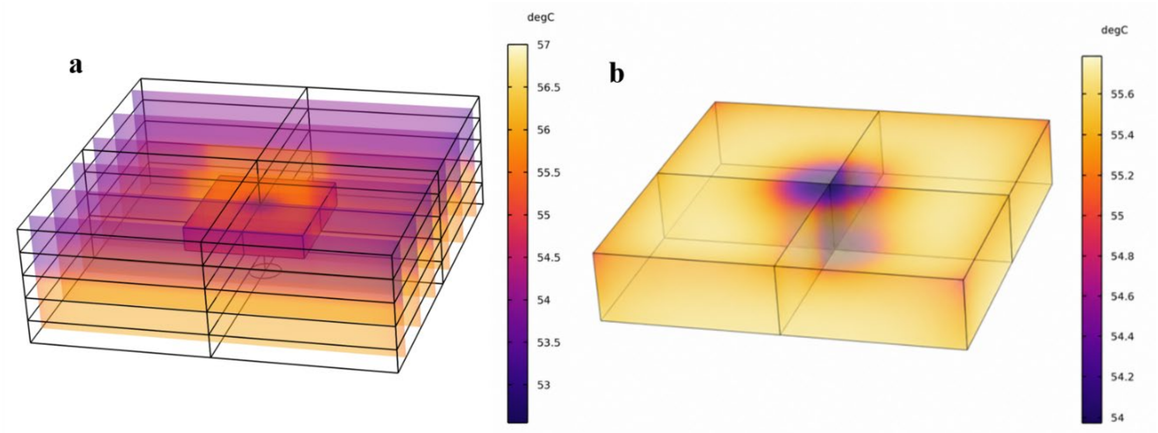

Figure S3 A three-dimensional heat transfer model of a heating stage-homemade glass device. (a) The temperature field distribution of the heating stage and the overall structure of the glass device, (b) The temperature distribution of the water medium inside the device.

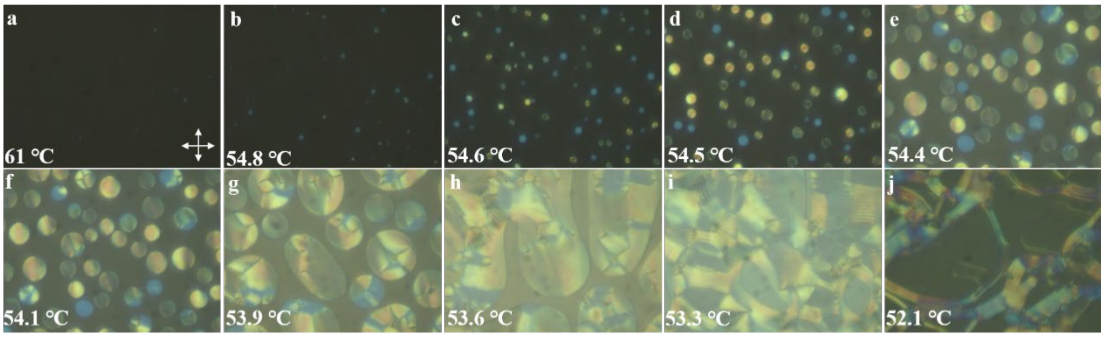

Fig S4 POM images of CLC film at a series of temperatures under the cooling process.
